# Temporal regulation of notch activation improves arteriovenous fistula maturation

**DOI:** 10.1186/s12967-022-03727-7

**Published:** 2022-11-23

**Authors:** Qunying Guo, Guang Chen, Hunter Cheng, Ying Qing, Luan Truong, Quan Ma, Yun Wang, Jizhong Cheng

**Affiliations:** 1 Department of Nephrology, Key Laboratory of Nephrology, The First Affiliated Hospital, Sun Yat-sen University, Ministry of Health and Guangdong Province, Guangzhou, China; 2 Section of Nephrology, Department of Medicine, Selzman Institute for Kidney Health, Baylor College of Medicine, Houston, TX 77030 USA; 3 Department of Radiation Oncology, The University of Texas MD Anderson Cancer Center, Houston, TX 77030 USA; 4 Department of Pathology, Houston Methodist Hospital, Houston, TX 77030 USA; 5 Department of Integrated Traditional Chinese and Western Medicine, Tongji Medical College, Huangzhong University of Science and Technology, Wuhan, China

**Keywords:** Arteriovenous fistula, Neointima formation, Chronic kidney disease, Vascular smooth muscle cell, Notch signaling

## Abstract

**Background:**

Arteriovenous fistula (AVF) maturation is a process involving remodeling of venous arm of the AVFs. It is a challenge to balance adaptive AVF remodeling and neointima formation. In this study we temporally controlled Notch activation to promote AVF maturation while avoiding neointima formation.

**Methods:**

Temporal Notch activation was controlled by regulating the expression of Notch transcription factor, RBP-Jκ, or dnMAML1 (dominant negative MAML2) in vascular smooth muscle cells (VSMCs). AVF mouse model was created and VSMC phenotype dynamic changes during AVF remodeling were determined.

**Results:**

Activated Notch was found in the nuclei of neointimal VSMCs in AVFs from uremic mice. We found that the VSMCs near the anastomosis became dedifferentiated and activated after AVF creation. These dedifferentiated VSMCs regained smooth muscle contractile markers later during AVF remodeling. However, global or VSMC-specific KO of RBP-Jκ at early stage (before or 1 week after AVF surgery) blocked VSMC differentiation and neointima formation in AVFs. These un-matured AVFs showed less intact endothelium and increased infiltration of inflammatory cells. Consequently, the VSMC fate in the neointima was completely shut down, leading to an un-arterialized AVF. In contrast, KO of RBP-Jκ at late stage (3 weeks after AVF surgery), it could not block neointima formation and vascular stenosis. Inhibition of Notch activation at week 1 or 2, could maintain VSMC contractile markers expression and facilitate AVF maturation.

**Conclusions:**

This work uncovers the molecular and cellular events in each segment of AVF remodeling and found that neither sustained increasing nor blocking of Notch signaling improves AVF maturation. It highlights a novel strategy to improve AVF patency: temporally controlled Notch activation can achieve a balance between adaptive AVF remodeling and neointima formation to improve AVF maturation.

**Translational perspective:**

Adaptive vascular remodeling is required for AVF maturation. The balance of wall thickening of the vein and neointima formation in AVF determines the fate of AVF function. Sustained activation of Notch signaling in VSMCs promotes neointima formation, while deficiency of Notch signaling at early stage during AVF remodeling prevents VSMC accumulation and differentiation from forming a functional AVFs. These responses also delay EC regeneration and impair EC barrier function with increased inflammation leading to failed vascular remodeling of AVFs. Thus, a strategy to temporal regulate Notch activation will improve AVF maturation.

**Supplementary Information:**

The online version contains supplementary material available at 10.1186/s12967-022-03727-7.

## Introduction

Vascular access is the Achilles’ heel of patients undergoing hemodialysis. A functioning vascular access is necessary for the patients to receive successful life-sustaining hemodialysis. Arteriovenous fistula (AVF) is a surgical connection of an artery (such as radial artery) directly to a vein (cephalic vein), the venous limb remodels adequately and successfully to support repeated cannulations, acting as a bridge between blood and hemodialysis machine. KDOQI (Kidney Disease Outcomes Quality Initiative) vascular access guideline sets Fistula First as the goal for hemodialysis because the thrombosis rates, infection rates, access-related expenditures, and total healthcare expenditures all are lower for patients with fistulas than for those with either synthetic AV grafts or central venous catheters [1]. Unfortunately, about 40–60% of primary vascular accesses fail in two years and require medical interventions that cost of > $1 billion annually. Multicenter, randomized clinical trials targeting thrombosis have produced only a limited improvement in the patency of AVFs and AV grafts [2–8]. Therefore, uncovering cellular mechanisms and molecular pathways that regulate vascular remodeling could help develop clinical strategies to benefit maturation and patency of AVFs.

In AVFs, the vein anastomosis is subjected to increased blood flow/pressure. This causes mechanical stretching, which leads to the development of a dilated, thickened vein wall to withstand high hemodynamic forces and repeated cannulations. The venous outflow limb must dilate while remaining sufficiently pliable to maintain blood flow/pressure after moderate extracellular matrix deposition and cellular migration/proliferation. These components must be integrated to form a functional AVF [9–11]. AVF can fail if the venous arm does not remodel adequately (arterialization) to support hemodialysis or if the venous arm develops neointimal hyperplasia [12]. Excessive neointimal formation will narrow the lumen and block blood flow, causing AVF dysfunction and thrombosis. Hemodialysis Fistula Maturation Study Group reported the prevalence of stenosis detected on ultrasound was 14% at 1 day and increased to 30% from 2 to 6 weeks after creation of AVF. Notably, these postoperative fistulae venous stenosis causing by intimal hyperplasia significantly associates with fistula maturation failure [13]. Therefore, facilitating AVF adaptive remodeling while controlling neointima formation is a current challenge.

Understanding the cellular and molecular events during AVF remodeling will lead to solutions to a targeted therapeutic strategy to improve AVF patency. We have shown that VSMCs from anastomosed arteries are dedifferentiated and migrate into the venous anastomosis to form neointima in AVFs [14, 15]. Although multiple signaling pathways have been involved in regulating vascular remodeling [16], Notch signaling determines VSMC differentiation and artery formation during development [17]. Whether manipulating Notch signals can balance the lack of remodeling and neointima hyperplasia to improve AVF maturation has not been studied.

Notch and its ligands transduce signals between neighboring cells from signal-giving cells expressing Notch ligands to signal-receiving cells expressing Notch receptors. When a Notch ligand binds to Notch, the extracellular domain of the Notch receptor is cleaved, resulting in release of an intracellular Notch domain (abbreviated NICD). NICD can interact with RBP-Jκ, a primary transcription factor of Notch signaling [18]. NICD displaces co-repressors from RBP-Jκ, and replaces them with co-activators [e.g., master mind 1 (MAML1)]. The binding of MAML1 to RBP-Jκ results in transcription of target genes, Hes1/5, Hey2, and VSMC markers, Myh11 and α-SMA [19–23]. A dominant negative MAML1 (dnMAML1) can block Notch activation. Therefore in this study we studied the roles of Notch transcription factor (RBP-Jκ and MAML1) on VSMC differentiation and neointima formation during AVF remodeling. We used temporal controlled expression of RBP-Jκ or dnMAML1 to manipulate Notch activation in VSMCs to improve AVF adaptive remodeling and maturation.

## Methods

### Mice

All studies were approved by the Institutional Animal Care and Use Committee of Baylor College of Medicine in accordance with NIH guidelines. They were kept in a 12-h light/12-h dark cycle. Male wild type and mT/mG mice were obtained from Jackson Laboratory (Bar Harbor, Maine). RBP-Jκ-floxed mice were kindly provided by Dr. K. Susztak (Albert Einstein College of Medicine, NY). SMMHC-CreER^T2^ transgenic mice were obtained from Dr. S. Offermanns (Max-Planck-Institute for Heart and Lung Research, Bad Nauheim, Germany) and used as described [15]. To generate mice with KO of RBP-Jκ in VSMCs, mice with a floxed RBP-Jκ allele were bred with SMMHC-ERCre mice. After a backcross, RBP-Jκ^f/f^/SMMHC-ERCre^+^ (VSMC^*RBP-J*κ *KO*^) mice were obtained. Male mice from VSMC^*RBP-J*κ *KO*^ mice and littermate controls mice were studied. RBP-Jκ deletion was induced by Tamoxifen injection (2 mg/mouse/day *i.p.* for 5 days).

### *Creating CKD* mouse model

CKD in mice was induced by subtotal nephrectomy under anesthesia (ketamine, 125 mg/kg BW and xylazine, 6.4 mg/kg BW) [24, 25]. Mice (aged 10–12 wk and matched for body weight) underwent two-step subtotal nephrectomy. First, ~ 2/3 of the left kidney was removed. Then, the mice were given 1 dose of slow release buprenorphine (1 mg/kg BW by subcutaneous injection) before the surgery. To reduce mortality and limit kidney hypertrophy, mice were fed 6% Protein Rodent Diet Chow (Harlan Teklad, Madison, WI, USA) ad libitum. One week later, the right kidney was removed from mice while anesthetized. After one week of recovery, mice with CKD were fed 40% protein chow and compared to sham-operated control mice. BUN and serum creatinine levels were measured. After 2–3 weeks, AVFs were created in control and CKD mice. Operators for all animal experiments were blinded to group allocation during all analytical procedures.

### Mouse AVF Model

AVF was created in each mouse as described [26]. Briefly, the left internal jugular vein was isolated and its distal end, and the proximal common carotid artery was ligated below its bifurcation [27]. An end-to-end anastomosis was created using 12–0 nylon suture with an interrupted stitch. After unclamping, patency was confirmed visually. The venous side became inflated after releasing the clamp at the common carotid artery, indicating the success of the AVF surgery (Additional file 1: Fig. S1). The mice were kept warm after surgery, and the analgesic (buprenorphine) was given before surgery. At different time points after surgery, anesthetized mice were euthanized by perfusing the left ventricle with 10 ml of PBS and 10% formalin for 10 min. AVFs were dissected and fixed. Sections were obtained from the venous anastomosis to 1 mm from the AVF and morphology of AVFs was examined after paraffin-embedding. The area of neointima and media were defined as the regions between the lumen and the adventitia. The vessel wall thickness was measured by NIS-Elements BR 3.0 program (Nikon, Tokyo, Japan) as area of the vessel minus that of the lumen. Five slides of cross sections were obtained by selecting the first of every 10 sections from each AVF and were used to evaluate neointima formation in AVFs. AVF’s from hemodialysis patients were collected and evaluated as described [28].

### Evans blue examination of endothelial barrier function of AVFs

At 4th week after AVF surgery, the mice were anesthetized with ketamine and xylazine. Total 50 μl of 5% Evans blue (Sigma-Aldrich, E2129) diluted in saline was injected into the right external jugular vein and kept for 5 min. And then the mice were euthanized by perfusion of the right ventricle with 10 ml PBS, and then 10 ml 10% formalin and kept for 10 min, respectively. After the mice were euthanized, AVFs were removed and photographed. The area of Evans Blue staining was semi-quantified using ImageJ software as previous reports [26].

### Stimulating dnMAML1 overexpression in mice

We used the TetOn/rtTA (reverse tetracycline transactivator) inducible system and Flox-Cre recombination techniques to create SMC specific conditional inducible dnMAML1 transgenic mice. To breed these mice, three strains of mice were required (Fig. 7A): (1) SM22-Cre mice (Jackson lab), that constitutively expressed Cre recombinase, driven by the tissue specific promoter in SMCs; (2) rtTA-EGFP transgenic mice (Jackson lab), in which the expression of floxed-*rtTA* is under the control of the ubiquitously expressed *ROSA26* promoter; a floxed stop cassette present between the *ROSA26* promoter and *rtTA,* confined rtTA expression to the cells in which Cre recombinase was present. Furthermore, an internal ribosome entry site (IRES) followed by the *EGFP* gene was located downstream of *rtTA*, thereby allowing tracking of the rtTA-expressing cells by EGFP expression; and (3) the TetO-dnMAML1-GFP transgenic mice (Jackson lab), driven by the tetracycline inducible promoter (*tetO).* The genotyping was performed according to the protocol provided in Jackson Laboratory. The resulting line hereafter was referred to as idnMAML1/VSMC mice. Transgenic littermates were treated with Dox-containing water (0.5 mg/ml; Sigma Chemicals, with 5% sucrose added). dnMAML negative mice that were treated with Dox were used as control. The expression of dnMAML1 was determined by Western blots.

### Reagents and virus

Penicillin, streptomycin, DMEM, FBS, fluorescent-680 (Cat. number: A-10038) or 800 (Cat. number: A-32808) secondary antibodies for Western blotting, and all secondary antibodies for immunefluorescent staining were obtained from Invitrogen (Invitrogen Life Technologies; Carlsbad, CA). Human TGF-β1 was obtained from R&D (Minneapolis, MN). Antibodies against calponin (Cat. number: C2687), α-SMA-FITC (Cat. number: F3777), α-SMA (Cat. number: A5228), calponin (Cat. SAB420710), and β-actin (Cat. number: A5441) were from Sigma-Aldrich (Louis, MO). Antibodies against PCNA (Cat. number: sc-7907), ICAM (Cat. number: sc-71303), IL-1β (Cat. number: sc-12742), transgelin (Cat. Sc-18513), and GAPDH antibodies (Cat. number: sc-32233) were from Santa Cruz Biotechnology (Santa Cruz, CA). Antibodies against PCNA (Cat. number: ab92552), rabbit anti-α-SMA (Cat. number: ab5694), GFP (Cat. number: ab6556), F4/80 (Cat. number: ab6640) were from Abcam (Cambridge, MA). CD45 antibody (Cat. number: 553076), and VE-cadherin (Cat. number: 550548) were from BD Pharmingen (San Jose, CA). Antibodies against PECAM (CD31) (Cat. number: DIA-310-M) and Mac2 (Cat. number: CL8942AP) were from Cedarlane (Burlington, NC), Control rabbit IgG (Cat. Number: S-5000) was from Vector Labs (Burlingame, CA). The detailed information about antibodies used in this study was listed in Additional file 2: Table S1. 4′,6-diamidino-2-phenylindole (DAPI) was from Southern Biotech (Birmingham, AL). The packaging vectors for lentivirus were purchased from Addgene (Cambridge, MA). The lentivirus-infected cells were selected by puromycin treatment.

### Mouse VSMCs isolation and cell culture

Mouse VSMCs were isolated as previously described [29, 30] and cultured in DMEM supplemented with 20% heat inactivated fetal bovine serum (FBS; Hyclone, Logan, UT, USA), 100 μg/ml penicillin, and 100 μg/ml streptomycin (Invitrogen Life Technologies, Carlsbad, CA, USA).

### Histology and immunohistochemistry

For histological analyses, mice were perfused through the left ventricle, and slides of AVFs were prepared as described [15]. Immunohistochemical staining and H and E-stained sections from AVFs of 5 mice in each group were examined by a pathologist who was blinded to treatment. For double immunofluorescence staining, primary antibodies were added, followed by fluorescent secondary antibodies; DAPI was used to stain nuclear DNA. Isotype-matched IgG was used as negative controls. To capture images, the Nikon Eclipse 80i fluorescence microscope (Melville, NY) was used. The areas of positive signal were measured using the NIS-Elements BR 3.0 program. Images (× 400) for each section were analyzed in a blinded manner and quantified using Image-Pro Plus software (Media Cybernetics, Silver Spring, Md., USA).

### Western blot analysis

Mouse SMCs were lysed in RIPA buffer, and ~ 20 μg of proteins were separated by SDS-PAGE. After transferring to NC membranes, antibodies were added [31].

### Statistical analysis

All experiments were performed with at least 3 independent biological or experimental replicates. Statistical analyses were performed in Graphpad Prism Version 8. Scatter dot-plots and error bars represent the mean ± SEM or median with interquartile range. Significant differences were determined by Mann–Whitney test, Student’s t-test (unpaired), and one-way or two-way ANOVA. Statistical tests are described in each figure legend. Differences were considered statistically significant at *P*-value < 0.05.

## Results

### KO of RBP-Jκ blocks VSMC accumulation and AVF maturation

**Fig. 1:**
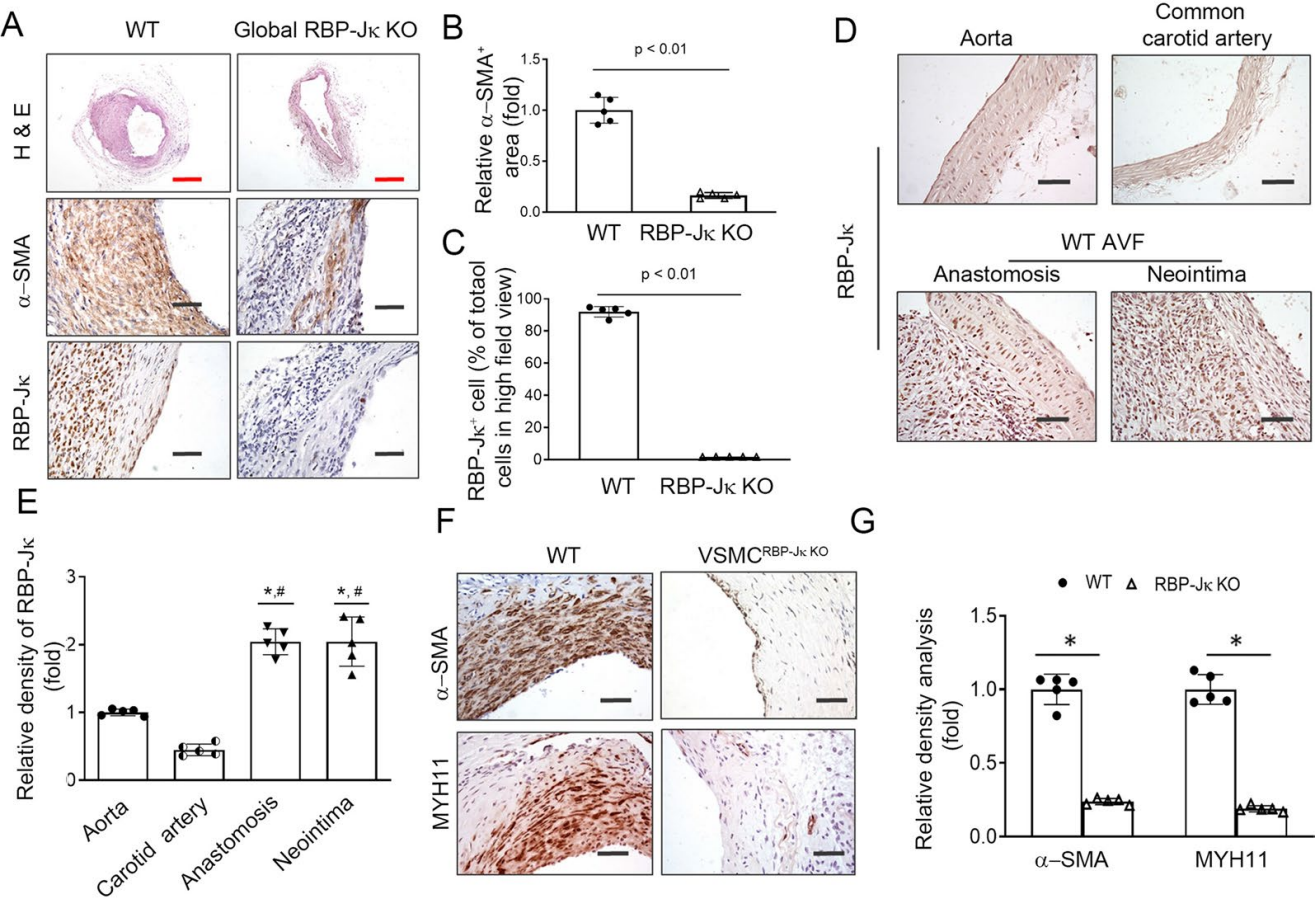
KO of RBP-Jκ blocks neointima formation and the expression of VSMC markers. **A**–**C** AVFs were created in WT and RBP-Jκ^f/f^/ELLA-ERCre^+^ mice after tamoxifen induction. Representative images show H & E and immunostainings of the α-SMA and RBP-Jκ (**A**). **B** and **C** Density analysis of the expression of α-SMA and RBP-Jκ in AVFs that were created in WT and global RBP-Jκ KO mice (n = 5, *, *p* < *0.05 vs.* WT controls; Mann–Whitney test was used for statistical analysis; black scales = 50 µm; red scales = 200 µm). **D** and **E** Representative images and densitometry analysis of the expression of RBP-Jκ in arteries and AVFs that were created in WT mice (n = 5, *p < 0.05 compared with aorta group; #*p* < 0.05 compared with carotid artery group; one-way ANOVA was used for statistical analysis). **F** and **G** AVFs were created in WT and VSMC^RBP−Jκ KO^ mice after tamoxifen induction. Representative images show α-SMA immunostainings of the AVFs created in uremic WT and VSMC^RBP−Jκ KO^ mice (n = 5, *p < *0.05 vs.* WT controls; Mann–Whitney test was used for statistical analysis; black scale bars = 50 µm; red scale bars = 200 µm)

To determine how Notch signaling regulates AVF maturation, we first used inducible whole body RBP-Jκ KO mice to create AVFs. Since RBP-Jκ is the transcription factor for all 4 Notch receptors, KO of RBP-Jκ will terminate all canonical Notch-dependent signaling (Additional file 1: Fig. S2). RBP-Jκ floxed mice were bred with ELLA-ERCre mice to generate RBP-Jκ^f/f^/ELLA-ERCre^+^ mice. Ella promoter directs expression of ERCre in all cell types. After tamoxifen treatment for 5 consecutive days, CKD and AVFs were created in WT (RBP-Jκ^f/f^/ELLA-ERCre^−^ mice) and RBP-Jκ^f/f^/ELLA-ERCre^+^ mice. We found that global KO of RBP-Jκ significantly decreased the wall thickness of the AVFs (Fig. 1A and B). Immunostaining results confirmed that RBP-Jκ had been efficiently knocked out in > 95% of cells in AVFs (Fig. 1A). Global KO of RBP-Jκ dramatically decreased the expression of VSMC marker α-SMA (Fig. 1B and C). We found that there were more cells accumulated in anastomosis and neointima of AVFs *vs*. that in aorta or common carotid artery, and most of these cells were RBP-Jκ positive (Fig. 1D and E). The neointimal cells in AVFs were also positive for contractile markers, including α-SMA, MHY11, transgelin, and calponin (Additional file 1: Fig. S3).

To further specify the function of RBP-Jκ in regulating VSMC phenotype and neointima formation in AVFs, we created AVFs in VSMC specific RBP-Jκ KO mice by using smooth muscle myosin heavy chain (SMMHC)-driven ERCre (SMMHC-ERCre). After tamoxifen treatment daily for 5 consecutive days, RBP-Jκ was KO in most of the VSMCs (> 95%). As shown in Fig. 1F and G, inducible KO of RBP-Jκ specifically in VSMCs completely blocked the expression of VSMC markers (α-SMA and Myh11) in neointima in AVFs created in uremic mice. These results demonstrate that Notch signaling is required for AVF remodeling. KO of RBP-Jκ-dependent signaling blocks VSMC marker expression, inhibiting AVF maturation.

### Loss of VSMC accumulation delays EC regeneration and increases inflammation in AVFs

**Fig. 2:**
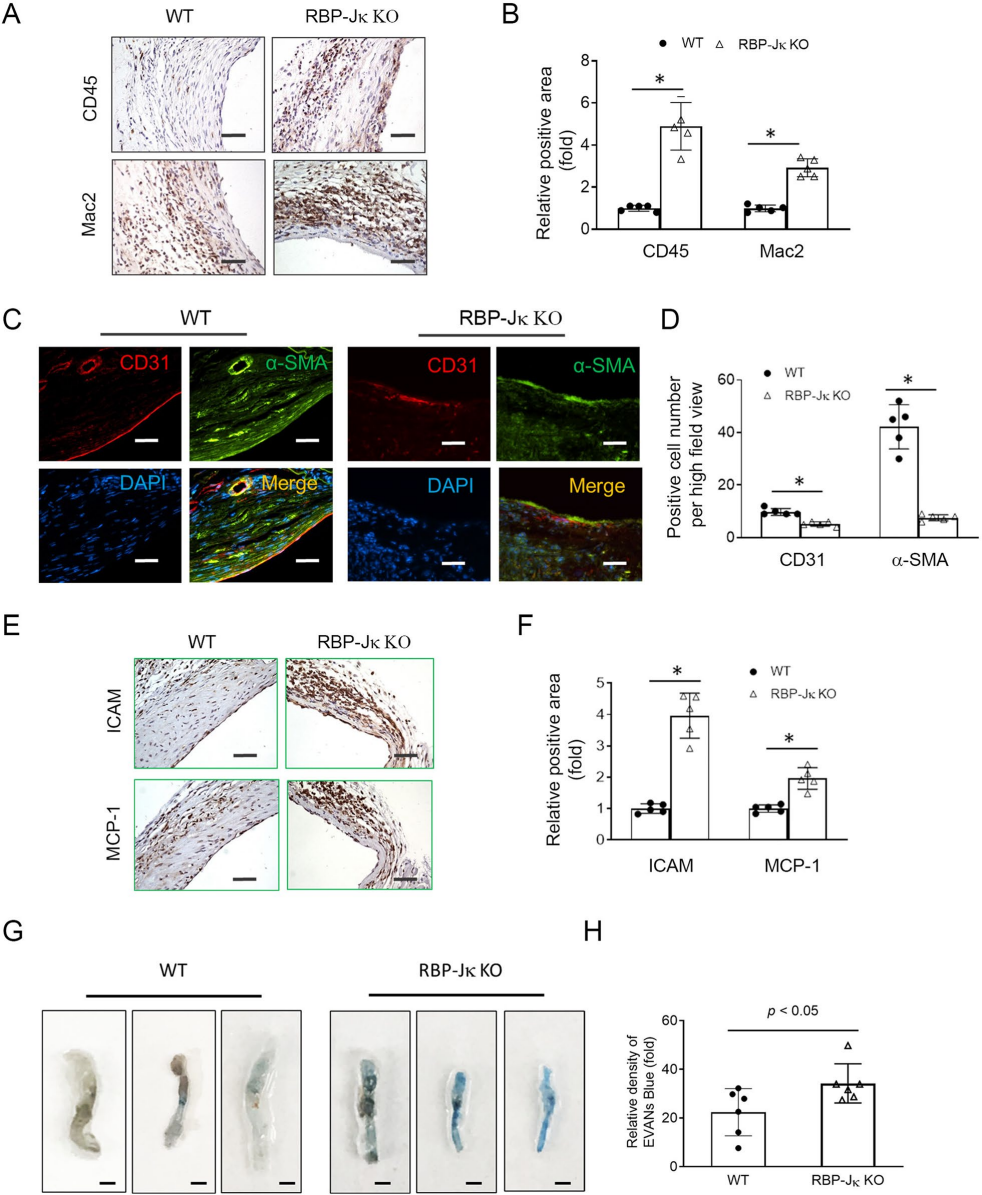
Loss of AVF remodeling is associated with delayed EC regeneration and increased inflammation. **A** and **B** Representative images of immunostaining of CD45 and Mac2 in AVFs created in uremic WT and VSMC^RBP−Jκ KO^ mice. The density analysis is shown in **B**. **C** and **D** The expression of EC marker CD31 was determined by immunostaining in 1 month AVFs created in WT and VSMC^RBP−Jκ KO^ mice. **E** and **F** The expression of adhesion molecule ICAM and cytokines (MCP-1) was determined by immunostaining 1 month AVFs created in WT and VSMC^*RBP−J*κ *KO*^ mice (**E**). The density analysis is shown in **F**. (n = 5; *p < 0.05; t-test was used for statistical analysis; Scale bars = 50 μm). **G** and **H** EVAN’s Blue staining of 1 month AVFs created in WT and VSMC^*RBP−J*κ *KO*^ mice (**G**). The density analysis is shown in **H**. (n = 6; *p < 0.05; t-test was used for statistical analysis; Black and white scale bars = 100 mm)

Accompanied with the loss of VSMC accumulation, there was an increased number of inflammatory cells, including CD45^+^ monocytes and Mac2^+^ macrophages, in AVFs created in VSMC^RBP-Jκ KO^ mice *vs.* that in WT mice (Fig. 2A and B). Since EC barrier dysfunction could lead to infiltration by inflammatory cells, we determined the expression of EC markers in AVFs. We found that the expression of EC marker (CD31) was decreased, and the integrity of the endothelial layer was damaged in AVFs created in VSMC^RBP-Jκ KO^ mice *vs.* that in WT AVFs (Fig. 2C and D). Moreover, the adhesion molecule ICAM and cytokines MCP-1 were increased in AVFs from VSMC^RBP−Jκ KO^ mice *vs.* that in WT AVFs (Fig. 2E and F). Consistently, Evans Blue staining showed that the stronger blue signals were found in AVFs created in VSMC^RBP-Jκ KO^ mice *vs.* that in WT AVFs (Fig. 2G and H). These results indicate that the absence of VSMC accumulation, due to inhibition of Notch-RBP-Jκ signaling in VSMCs, compromises the EC barrier function and leads to increased inflammation in AVFs.

### KO of RBP-Jκ does not block VSMC dedifferentiation in the anastomosis of AVFs

**Fig. 3:**
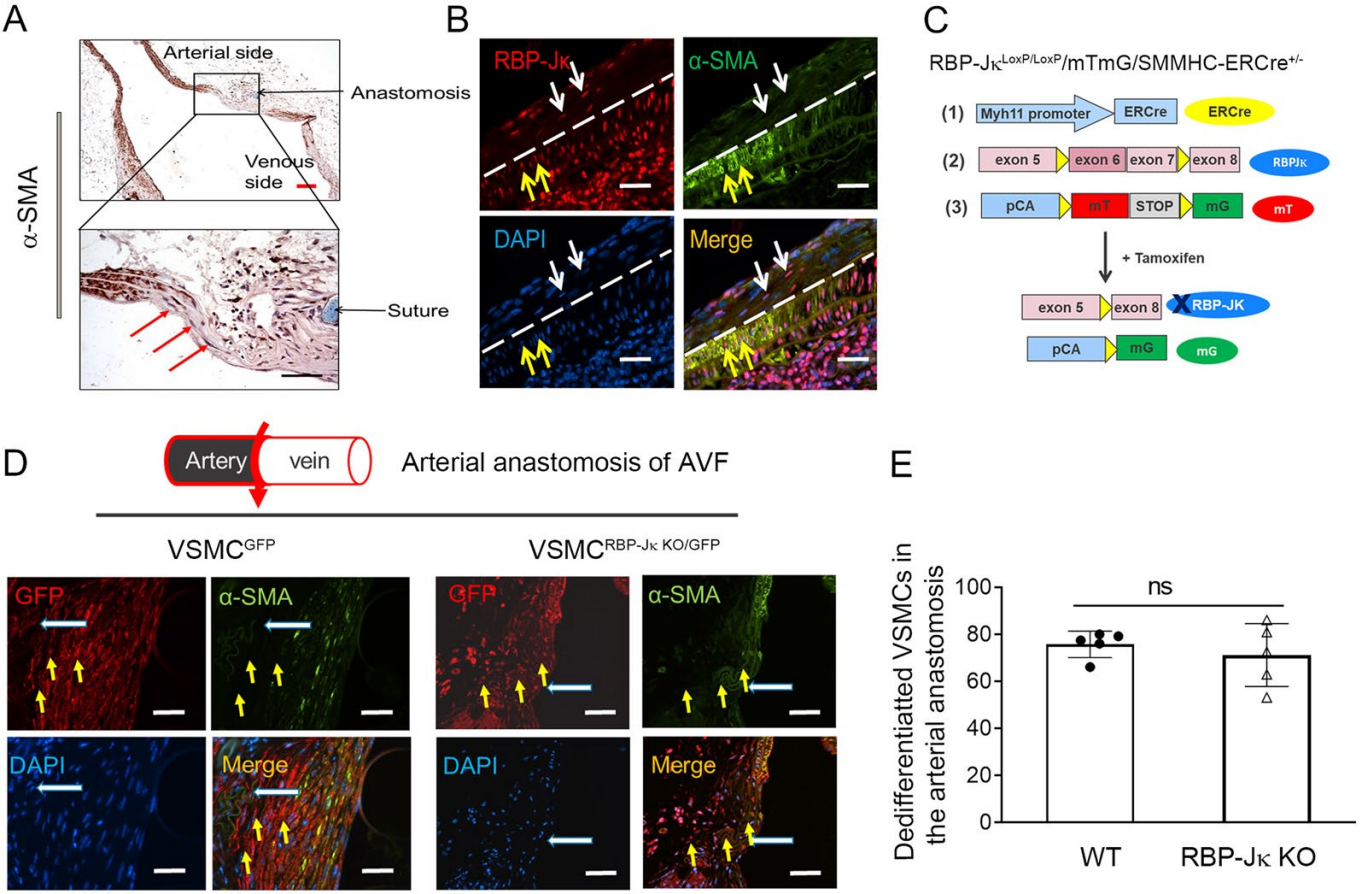
Notch signaling does not block VSMC dedifferentiation in AVFs. **A** Representative longitudinal images of α-SMA staining of 1 month AVFs that were created in WT mice. The red arrows point to the dedifferentiated VSMCs at AVF anastomosis. **B** The presence of RBP-Jκ in AVF anastomosis did not block VSMC dedifferentiation. Representative double immunostainings of RBP-Jκ and α-SMA are shown. Yellow arrows point to RBP-Jκ^+^/α-SMA^+^ cells and white arrows point to RBP-Jκ^+^/α-SMA^−^ cells. **C** Schematic of VSMC labeling in transgenic mice. **D** and **E** Representative images of double immunostaining of GFP and α-SMA in AVFs that were created in uremic VSMC^GFP^ and VSMC^RBP−Jκ KO/GFP^ mice (**D**). The number of GFP^+^/α-SMA^−^ cells (dedifferentiated VSMCs) was counted in the two groups (**E**). (n = 5; *p < 0.05; Mann–Whitney test was used for statistical analysis; all scale bars = 100 μm)

To understand how deficiency of Notch signaling in VSMCs blocks VSMC dedifferentiation, we investigated the dynamic phenotype switch of VSMCs during AVF remodeling. Longitudinal analysis of AVF created in WT mice showed the disappeared expression of VSMC marker (α-SMA) at arterial anastomosis in AVFs, indicating the dedifferentiation of VSMCs (Fig. 3A). These results are consistent with our previously report that VSMCs from anastomosed arteries migrate and contribute to neointima formation in AVFs [15, 32]. Thus, we hypothesize that Notch signaling could be essential in regulating the phenotypes of these dynamic VSMCs appeared during AVF remodeling.

To test our hypothesis, we firstly determined whether RBP-Jκ is related with VSMC dedifferentiation in AVFs. In AVFs created in WT mice, we found that expression of VSMC marker α-SMA disappeared in the neoinitma and in parts of the media area in the arterial side near the anastomosis, where the white dash line was used to indicate the neointima (upper side) and media layer (lower side) (Fig. 3B). The expression levels of RBP-Jκ had no differences between non-differentiated VSMCs (RBP-Jκ^+^/α-SMA^+^ double positive, the yellow arrows pointed cells) and dedifferentiated VSMCs (RBP-Jκ^+^/α-SMA^−^, the white arrows pointed cells) in AVF anastomosis created in WT mice (Fig. 3B).

To confirm if these α-SMA negative cells were derived from VSMCs, VSMCs were labeled with GFP in VSMC reporter mice that were created by breeding mTmG mice with SMMHC-ERCre mice (VSMC^GFP^ mice) (Fig. 3C). After tamoxifen induction, VSMCs and their lineages were labeled with GFP in VSMC^GFP^ mice (Additional file 1: Fig. S4). After breeding VSMC^GFP^ mice with RBP-Jκ^f/f^ mice, the VSMCs which were deficient of RBP-Jκ were labeled by GFP (VSMC^RBP−Jκ KO/GFP^ mice) (Fig. 3C). Ten days after the last dose of tamoxifen treatment, AVFs were created in uremic VSMC^GFP^ mice and VSMC^RBP−Jҡ KO/GFP^ mice. After 1 month, AVFs were collected and the cells in the anastomoses were stained to determine VSMC differentiation status. The GFP labeled VSMCs lost expression of α-SMA in the anastomoses in AVFs created in both VSMC^GFP^ mice and VSMC^RBP−Jκ KO/GFP^ mice (Fig. 3D). The numbers of dedifferentiated VSMCs were similar between the AVFs that were created in VSMC^GFP^ mice with RBP-Jκ^f/f^ mice (Fig. 3E). These results indicate that KO of RBP-Jκ does not block VSMC dedifferentiation in anastomosis of the AVFs.

### RBP-Jκ KO in VSMCs blocks neointimal VSMC differentiation and AVF maturation

**Fig. 4:**
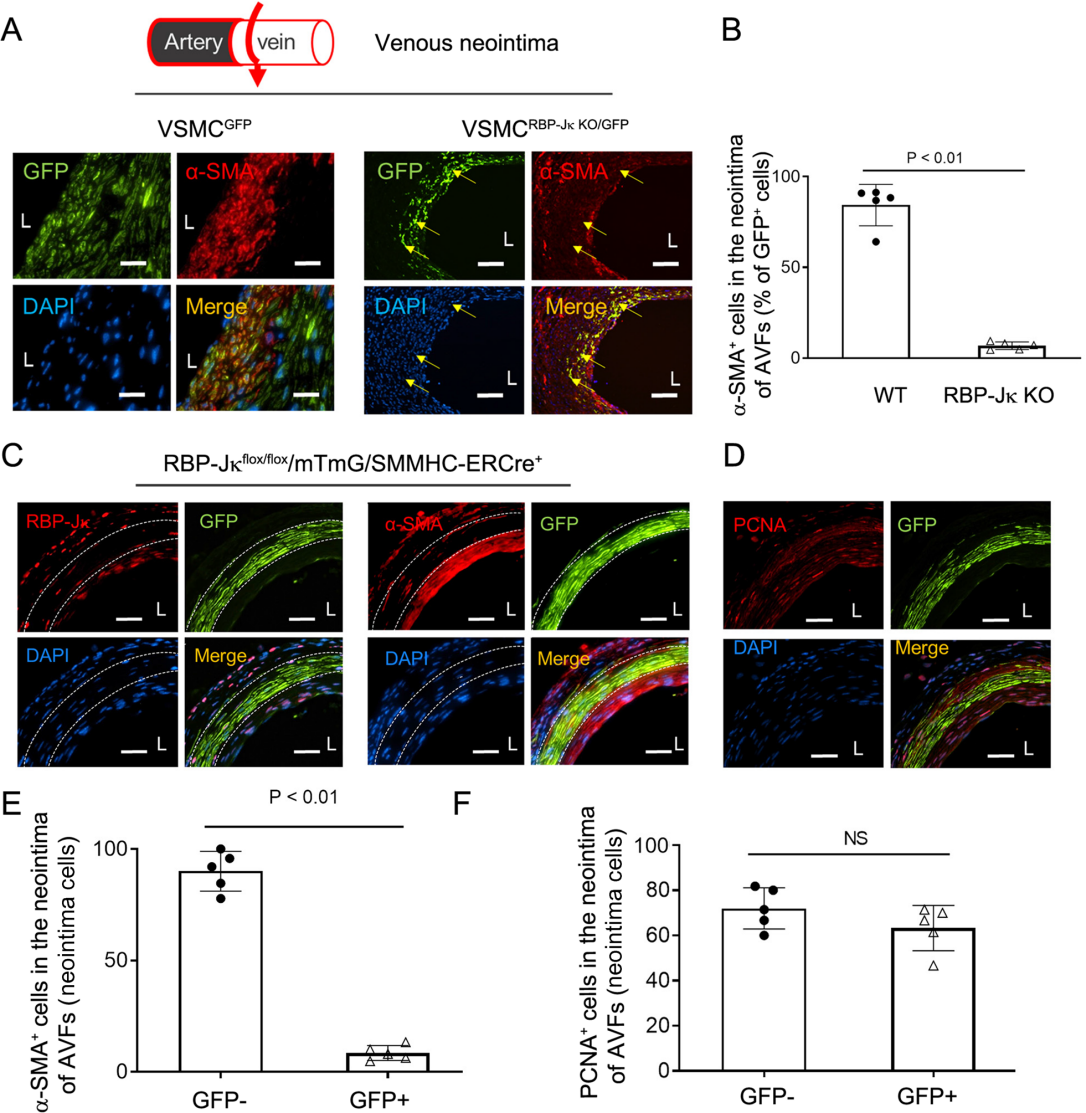
RBP-Jκ KO in VSMCs blocks neointimal VSMC differentiation and prevents AVF maturation. **A** AVFs were created in VSMC^GFP^ and VSMC^RBP−Jκ KO/GFP^ mice 10 days after last dose of tamoxifen treatment (i.p., 80 mg/Kg. bw for 5 consecutive days). Representative images of double immunostaining of GFP and α-SMA were presented (**A**) and the GFP^+^/α-SMA^+^ cells were counted and calculated (**B**). **C** and **D** AVFs were created in VSMC-GFP-RBP-Jκ KO (partial KO) mice after single tamoxifen treatment (i.p., 80 mg/Kg. bw). The 1 month AVFs were immunostained with GFP/RBP-Jκ (C), GFP/α-SMA (C), and GFP/PCNA (D). **E** The percentage of α-SMA^+^ cells in GFP^+^ cells (representing KO of RBP-Jκ in VSMC differentiation) *vs*. α-SMA^+^ cells in GFP^−^ cells in the neointima (represent RBP-Jκ positive SMCs) in Panel C were calculated. **F** The percentage of PCNA positive cells in both GFP^+^ and GFP^−^ cells in Panel D were calculated. (n = 5; *p < 0.05; Mann–Whitney test was used for statistical analysis; white scale bars = 50 μm)

VSMC reporter mice were used to evaluate the role of RBP-Jκ in VSMC differentiation from dedifferentiated VSMCs in neointima in AVFs. The GFP labeled VSMCs were α-SMA positive in the neointima of AVFs that were created in VSMC^GFP^ mice (Fig. 4A). However, in AVFs that were created in VSMC^RBP−Jκ KO/GFP^ mice, the GFP labeled VSMCs were α-SMA negative (yellow arrow-pointed neointima cells in Fig. 4A and B), indicating that dedifferentiated VSMCs (GFP^+^/α-SMA^−^) cannot regain the differentiation potential to become terminal differentiated VSMCs (expressing contractile markers) when RBP-Jκ is deficient.

To further confirm this results, we compared the differentiation status between VSMCs with or without RBP-Jκ in same AVFs. To induce RBP-Jκ KO only in part of VSMCs in VSMC^RBP-Jκ KO/GFP^ mice, we treated mice with one dose injection of tamoxifen. AVFs were created in VSMC^RBP-Jκ KO/GFP^ mice after only one dose of tamoxifen injection. The RBP-Jκ were knocked out in ~ 40% of VSMCs, which were labeled by GFP (Fig. 4C). These GFP positive VSMCs did not express the VSMC marker α-SMA (Fig. 4C); in contrast, the GFP negative cells (RBP-Jκ positive) regained the expression of α-SMA (Fig. 4C). The percentage of α-SMA^+^ cells in total GFP positive cells (representing VSMCs with RBP-Jκ KO) was significatnly lower than that in GFP negative cells (representing WT VSMCs) (Fig. 4E). The number of PCNA positive cells was similar in GFP^+^ and GFP^−^ cells in the neoinitma in AVFs created in VSMC^RBP-Jκ KO/GFP^ mice (Fig. 4D and F), suggesting that KO of RBP-Jκ controls VSMC fate but has no markable effects on VSMC proliferation during neointima formation.

### RBP-Jκ is not required to maintain VSMC phenotype in blood vessels for adult mice

**Fig. 5:**
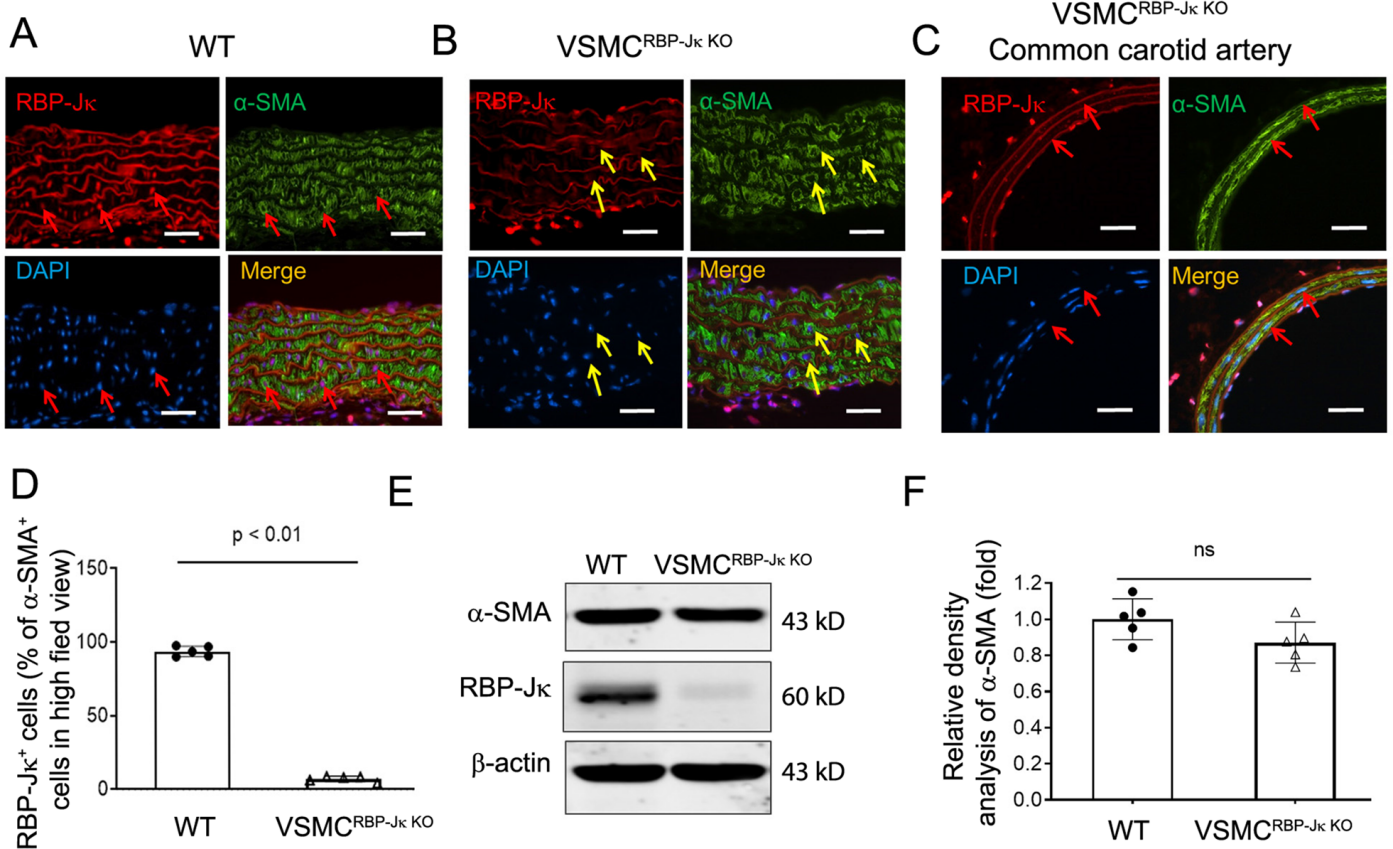
RBP-Jκ is not required to maintain VSMC phenotype in mature arteries. **A** and **B** WT and VSMC^RBP−Jκ KO^ mice were treated with tamoxifen (i.p., 40 mg/kg. b.w.) for 5 days. The expression of RBP-Jκ and α-SMA were determined in the aorta of WT (**A**) and VSMC^RBP−Jκ KO^ mice (**B**). The arrows point to the nuclear of the media VSMCs. **C** The expressions of RBP-Jκ and α-SMA were also determined in common carotid artery of VSMC^RBP−Jκ KO^ mice. The arrows point to the nuclear of the media VSMCs. Notably, the expressions of RBP-Jκ in endothelial cells and adventitial cells were not affected. **D** The number of RBP-Jκ in α-SMA^+^ cells was counted in the arteries from WT and VSMC^RBP−Jκ KO^ mice. **E** and **F** Representative Western blotting and densitometry analysis of the expression of VSMC marker (α-SMA) from aorta isolated from WT and VSMC^RBP−Jκ KO^ mice. (n = 5; *p < 0.05; Mann–Whitney test was used for statistical analysis; white scale bars = 50 µm)

Since deficient of RBP-Jκ blocked the expression of contractile marker expression in neointimal VSMCs, we investigated if inducible KO RBP-Jκ would block the expression of the contractile markers in VSMCs in the arteries in adult mice. Our results showed that VSMC-specific KO of RBP-Jκ did not block VSMC marker expression in aorta (Fig. 5A and B) or in common carotid artery after 1 month (Fig. 5C). The efficiency of RBP-Jk KO in VSMCs was > 90% (Fig. 5D). In a 6 month period, there was no significant difference in the expression of α-SMA in arteries in RBP-Jκ KO mice vs that of WT mice, though the level of α-SMA was decreased in RBP-Jκ KO mice (Fig. 5E and F). These results demonstrate that RBP-Jκ is required for VSMC fate determination, but not required for maintaining VSMC fate once the VSMCs have terminally differentiated.

### Temporal and VSMC-Specific KO of RBP-Jκ improves AVF maturation

The above results demonstrate that Notch/RBP-Jκ activation is required for AVF maturation. Loss of Notch signaling blocks VSMC differentiation and migration and impairs AVF thickening. On the other hand, Notch/RBP-Jκ signaling is not required for maintenance of VSMC phenotype once the VSMC fate has been triggered/established. Thus, we propose that temporal regulation of Notch/RBP-Jк signaling may promote AVF maturation while preventing neointima hyperplasia.

**Fig. 6:**
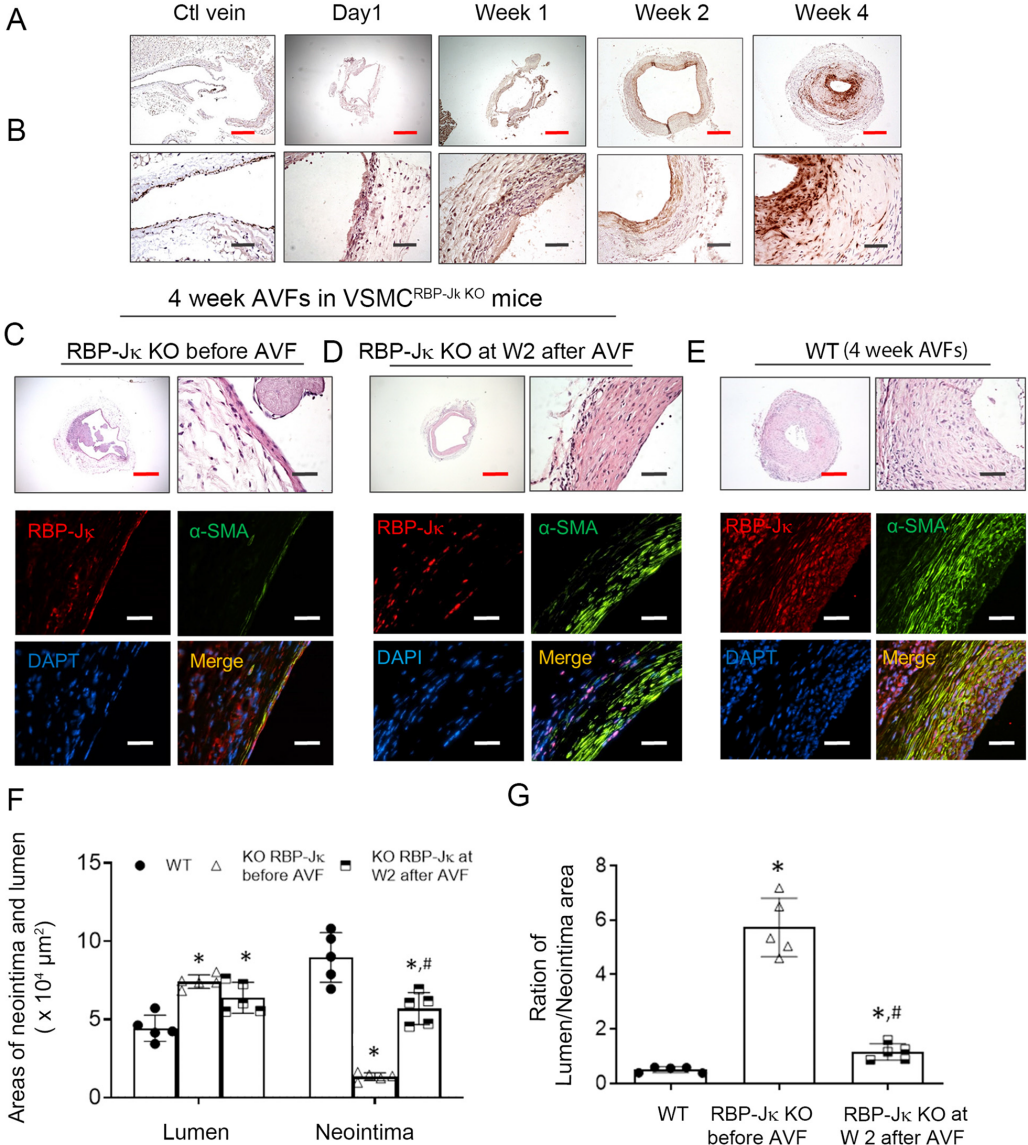
Temporal and VSMC-Specific KO of RBP-Jκ Improves AVF Maturation. **A** and **B** Time course of the neointima formation in AVFs. CKD and AVFs were created in mice. AVFs were collected at indicated time after surgery. α-SMA immunostaining was performed. The representative images were presented (n = 3). **C**–**E**. CKD model and AVFs were created in control mice (**E**); or created in VSMC^RBP−Jκ^ KO mice that were treated with tamoxifen before surgery (**C**) or 2 weeks after the AVF surgery (**D**). The H & E and immunostaining of RBP-Jκ and α-SMA staining were determined (**C**–**E**). **F** and **G** The areas of the neointima and lumen, as well as the ratio of neointima to lumen in AVFs created in Panel C–E were measured and calculated. (n = 5, *p < 0.05 compared with WT; #*p* < 0.05 compared with KO RBP-Jκ before AVF group; one-way ANOVA was used for statistical analysis; black and white scale bars = 50 µm; red scale bars = 200 µm)

To test this hypothesis, we need to find the “timing” of when the VSMCs in venous arm of AVFs begin expressing contractile proteins, so that Notch signaling can be blocked to prevent further Notch activation but without blocking VSMC fate in neointimal cells. AVFs were collected at different time points to discover the time point. There was no detectable expression of VSMC marker α-SMA and neointima formation in 1 week AVFs (Fig. 6A and B). However, plenty of α-SMA positive cells were detected in 2 week AVFs, indicating that these neointima cells had differentiated into VSMCs (Fig. 6A and B). Importantly, at this stage, there was limited neointima area in 2 week AVFs *vs.* that found in 4 weeks (Fig. 6A and B).

When tamoxifen treatment was given to induce KO of RBP-Jκ in VSMCs before surgery, there was almost no α-SMA^+^ VSMC accumulation in venous side of the AVFs that were created in VSMC^RBP-Jκ KO^ mice, thus no AVF maturation (middle panel in Fig. 6C). Next we determined if KO of RBP-Jκ 2 weeks after AVFs creation would inhibit neointima hyperplasia without blocking expression of VSMC markers and AVF maturation. As expected, when tamoxifen was given at two weeks after AVF creation in VSMC-RBP-Jκ KO mice, the VSMCs in neointima in AVFs were α-SMA positive in four weeks after AVF creation (Fig. 6D). Immunostaining results confirmed that the α-SMA^+^ cells were RBP-Jκ negative (Fig. 6D), indicating that KO of RBP-Jκ at this time point (2 weeks after AVF creation), when the VSMC fate has already been determined, could not block the expression of VSMC contractile markers. Compared with AVFs created in WT mice, there was decreased neointima formation and increased lumen area in VSMC-RBP-Jκ KO mice 2 weeks after AVFs creation (Fig. 6F and G). These results suggest that temporal KO of RBP-Jκ at 2 week after AVFs creation can improve AVF maturation while inhibiting neointima hyperplasia.

### Strategies of inducible switching notch signaling on and off in VSMCs in vivo

**Fig. 7:**
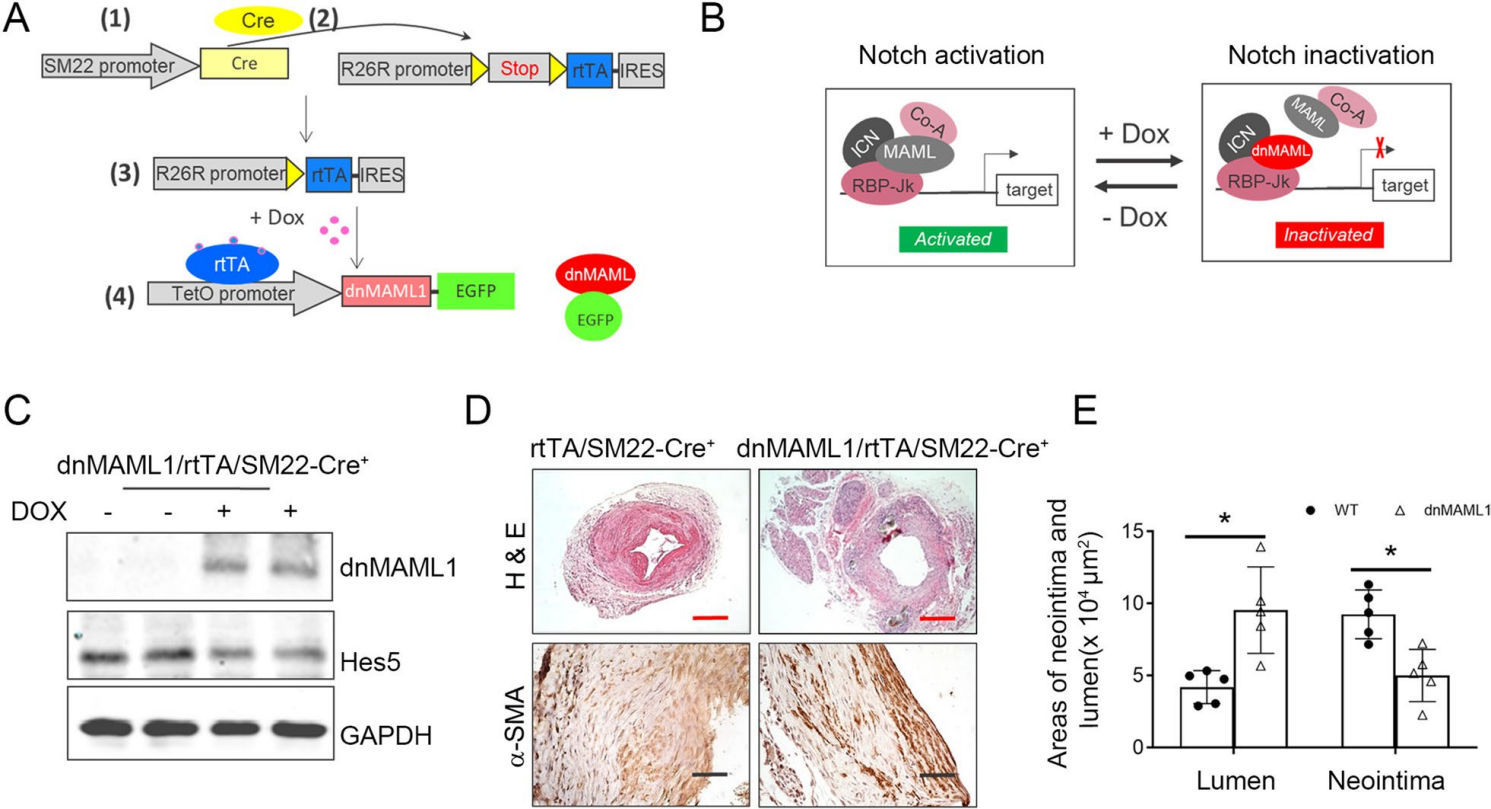
Strategies of Inducible Switching Notch Signaling On and Off in VSMCs in vivo*.* **A** Scheme of creating the transgenic mice with doxycycline-induced dnMAML expression in VSMCs. **B** dnMAML1 was induced in the presence of doxycycline and thus blocks Notch activation; halting doxycycline treatment for the triple transgenic mice VSMC^dnMAML1^ reactivates Notch. **C** VSMCs isolated from the VSMC^dnMAML1^ mice showed increased dnMAML1 expression and decreased Notch expression after Doxycycline treatment. **D** CKD and AVF were created in VSMC^dnMAML1^ mice. Doxycycline was administrated in week 2, and the representative images of H & E and α-SMA staining were shown. **E** The areas of lumen and neointima were calculated (n = 5; *p < 0.05; Mann–Whitney test was used for statistical analysis; black scales = 50 µm; red scales = 200 µm)

Since the Notch/RBP-Jκ pathway is important for VSMC differentiation, permanently knocking out this pathway could cause unexpected phenotype changes. To overcome this shortcoming, we developed a strategy to turn the Notch signaling “on” and “off” by removing or adding Doxycycline (Fig. 7A). We use a tetracycline (Tet)-controlled transcriptional activation to control dominant MAML1 expression. In the presence of doxycycline, reverse tetracycline-controlled transactivator (rtTA) binds with TetO (The TRE, tetracycline response element) and activates dnMAML1 expression. Removal of doxycycline terminates dnMAML1 expression and restores the Notch/RBP-Jκ responses (Fig. 7B). Doxycycline treatment for 5 days induced dnMAML1 expression but decreased the expression of Notch signal target, Hes5, in artery (Fig. 7C). Next, CKD and AVF were created in these VSMC^dnMAML1 OE^ transgenic mice. Doxycycline was added in the drinking water starting at two weeks after AVF surgery. This treatment significantly increased the lumen area and decreased the α-SMA^+^ cell accumulation without compromising the AVF maturation (Fig. 7D). Therefore, overexpression of DnMAML1 in VSMCs at two week after AVF creation decreased the neointima area *vs*. that in WT AVFs (Fig. 7E).

## Discussion

The vascular access is the lifeline for hemodialysis patients. Successful maturation following surgery requires functional and structural adaptations to arterial blood flows. Insufficient thickening of the AVF wall (maturation/remodeling) or excessive VSMC accumulation (neointima formation) leads to AVF dysfunction. In this study, we uncovered evidence showing that the Notch signaling pathway can be fine-tuned to improve AVF adaptive remodeling with limited neointima formation. Firstly, we found that the VSMCs at the anastomosis of AVFs became dedifferentiated. Secondly, the dedifferentiated, activated VSMCs migrated into the venous side and regained VSMC phenotype after expressing the contractile markers. Thirdly, inhibition of Notch signaling (KO of RBP-Jκ) blocks VSMC differentiation from dedifferentiated VSMCs in neointima and thus ablates AVF remodeling. We found that Notch signaling is required to initiate differentiation of VSMC progenitors into VSMCs, but it is not fully required to maintain VSMC phenotype. Fourthly, we showed that AVFs with insufficient venous wall thickening have endothelial barrier dysfunction and increased inflammation. Finally, we demonstrated that temporal control of Notch activation by KO of RBP-Jκ or OE of dnMAML1 improves AVF maturation.

VSMCs play an important role in neointima formation in AVF. Characterization of VSMC phenotype switching can provide insight to improve AVF maturation. Based on this study, there are at least two remarkable cellular events that occur after anastomosing end of vein to the artery. At beginning, the VSMCs in the anastomosis and in the venous arm of AVF are subjected to surgery trauma. Combined with the increased mechanical and shear stress after AVF operation, they become activated and dedifferentiated. These dedifferentiated VSMCs lose the expression of contractile VSMC markers (α-SMA and MHY11). They migrate, proliferate, and produce extracellular matrix in AVF, contributing to both vascular wall thickening and neointima formation. Then, the dedifferentiated VSMCs can regain the expression of VSMC markers in the neointima in AVFs. Dysregulation of these cellular events results in un-arterialized wall of AVF. As reported by Dardik, insufficient VSMC proliferation in the venous arm of the AVF will cause unsuccessful AVF maturation, while excessive VSMC accumulation will lead to AVF failure [12]. In addition, CKD uremic toxins promote VSMC hyperplasia and accelerate neointima formation leading to stenosis in AVFs. Thus, figuring out the molecular signaling pathways that are involved in VSMC phenotype switching in AVF will guide the development of targeted therapeutic strategy to properly regulate these cellular events and improve the AVF patency.

Notch signaling has been shown to regulate VSMC differentiation and artery formation during development. The VSMC recruitment into the arteries are severely blocked without Notch molecules [17]. Moreover, Notch signaling is one of the most important pathway leading to VSMC phenotype changes and accumulation in AVFs. We previously showed that sustained activation of Notch stimulates VSMC accumulation and neointima formation in AVF [33, 34]. Notch signaling promotes VSMC proliferation in response to cyclic stretch and CKD uremic toxins, which is closely associated with AVF failure [28, 35]. Superficially, we hypothesized that KO of Notch signaling could prevent neointima hyperplasia and improve AVF maturation. To our surprise, blockade of Notch signaling suppressed VSMC accumulation and completely blocked the expressions of contractile markers (MYH11 and α-SMA) in VSMCs, resulting in failure of VSMC accumulation and AVF remodeling and maturation. Others also reported that inhibition of Notch signaling blocks VSMC terminal differentiation from these SMC progenitors [36].

One of our interesting findings is that gaining VSMC markers (MYH11, ACTA2 and SM22) are essential for AVF function. Without expression of those contraction proteins, the dedifferentiated SMCs become more vulnerable to surrounding stimulus and lead to inflammation [37, 38]. Indeed, failed AVF remodeling impaired EC integrity and induced infiltration by inflammatory cells. In AVF created in RBP-Jκ global or VSMC-specific KO mice, the endothelial regeneration was delayed, and the leakage was observed indicating barrier dysfunction of the AVFs. This impaired EC barrier function could be caused by the lack of VSMC coverage. In AVF and AV graft models, a newly formed endothelium without VSMC coverage is vulnerable to stresses arising from arterial blood pressure [26, 39]. Consistent with this finding in AVF, we uncovered that KO of Jagged1 in VSMCs destabilize newly regenerated ECs in AV graft [40].

Another important finding of the study is that fine tuning Notch activation during AVF remodeling to manipulate the VSMC activation and differentiation could be the key to maintain this balance of AVF remodeling and neointima formation. We proposed that a temporally controlling Notch activation in VSMCs would both improve adaptive remodeling of AVF while preventing neointima hyperplasia. Importantly, Notch signaling is required to initiate VSMC differentiation, but not to maintain VSMC phenotype. We found that inhibition of Notch signaling after the appearance of VSMC markers in neointimal cells improved AVF remodeling and maturation. On the other hand, Doxycycline-induced “switch” to turn “On” and “Off” the Notch signaling by overexpressing dnMAML1 is even a better choice for temporal control of Notch activation. In this case, Notch activation can be inhibited or restored by using this “switch”, while Cre-mediated cleavage of foxed RBP-Jκ causes permanent deficiency of Notch activation, which has been shown to be related with other vascular diseases [41, 42].

VSMCs synthesize contractile markers to maintain artery homeostasis. Although Notch signaling is required to trigger progenitor cell differentiation into VSMCs, KO of RBP-Jκ did not significantly block the expression of VSMC contractile markers in cultured VSMCs or arteries of adult mice. It is possible that Notch stimulates and/or activates other signaling pathways to maintain VSMC phenotype in adult life. PDGFRβ, which has been reported to promote VSMC differentiation from stem cells, is one of the candidates that is regulated by Notch activation [43]. Others also showed that RBP-Jκ binding sites were present in the promoter of PDGFRβ [44]. Although other signals such as SRF/myocardin and Yes associated protein pathway also control PDGFRβ expression and VSMC differentiation [45–47], Notch is the master gene to determine VSMC fate.

## Conclusions

In summary, our results from this study demonstrate that Notch signaling plays a dominant role in determining VSMCs fate in AVF remodeling. Activated Notch signaling pathway promotes VSMC activation and differentiation, while deficiency of Notch impairs VSMC accumulation and induces EC barrier dysfunction and inflammation, leading to AVF maturation failure. Temporal control of Notch activation can improve AVF maturation. These findings could provide insight for developing therapeutic strategies by targeting the Notch signaling pathway to improve AVF maturation.

## Supplementary Information

**Additional file 1: Fig. S1.** Photograph of the common carotid artery and AVF after surgery. A. Right common carotid artery in normal mice. B. This picture shows the anastomosis of common carotid artery with internal jugular vein in AVF after surgery. The yellow arrow points to the sutures. The inflated jugular vein indicates the patency of the AVF. Scale bars = 1 mm. **Fig. S2.** Notch signaling conducts signals between neighboring cells. There are 5 Notch ligands including Jagged1/2, Dll1, 3 and 4; and 4 Notch receptors from 1 to 4. The interaction between Notch receptors and Notch ligands (Jagged/Delta) triggers 2 consecutive proteolytic cleavages by the ADAM10 metalloprotease and the γ-secretase complex. This generates Notch intracellular domain (NICD) which enters the nucleus and displaces corepressors and recruits the coactivator MAML1 and the acetyltransferase p300 to Notch transcription factor RBP-Jκ to initiate transcription of downstream signals. Canonical Notch signal can be blocked by KO of RBP-Jκ or overexpression of a dominant negative MAML1. **Fig. S3.** Contractile SMC markers are detected in mouse AVFs. Double immunofluorescent staining of α-SMA/transgelin (SM22) (A) and MYH11/calponin1 (CNN1) (B) were performed in 1 month AVFs. Scale bars = 50 μm. **Fig. S4.** VSMCs in common carotid artery were labeled with GFP in mTmG/SMMHC-ERCre+ mice after tamoxifen induction. Scale = 50 μm.

**Additional file 2: Table S1.** List of antibodies used in this study.

## Data Availability

All data generated or analysed during this study are included in this published article (and its supplementary information files).

## Abbreviations

AVF: Arteriovenous fistula
VSMC: Vascular smooth muscle cell
EC: Endothelial cell
