# Supplementary material for: Temporal regulation of notch activation improves arteriovenous fistula maturation
Source: J Transl Med. 2022 Nov 23;20:543. doi: 10.1186/s12967-022-03727-7 (PMC9682688; doi:10.1186/s12967-022-03727-7)
Supplement: Supplementary file 1 — Additional file 1: Fig. S1. Photograph of the common carotid artery and AVF after surgery. A. Right common carotid artery in normal mice. B. This picture shows the anastomosis of common carotid artery with internal jugular vein in AVF after surgery. The yellow arrow points to the sutures. The inflated jugular vein indicates the patency of the AVF. Scale bars = 1 mm. Fig. S2. Notch signaling conducts signals between neighboring cells. There are 5 Notch ligands including Jagged1/2, Dll1, 3 and 4; and 4 Notch receptors from 1 to 4. The interaction between Notch receptors and Notch ligands (Jagged/Delta) triggers 2 consecutive proteolytic cleavages by the ADAM10 metalloprotease and the γ-secretase complex. This generates Notch intracellular domain (NICD) which enters the nucleus and displaces corepressors and recruits the coactivator MAML1 and the acetyltransferase p300 to Notch transcription factor RBP-Jκ to initiate transcription of downstream signals. Canonical Notch signal can be blocked by KO of RBP-Jκ or overexpression of a dominant negative MAML1. Fig. S3. Contractile SMC markers are detected in mouse AVFs. Double immunofluorescent staining of α-SMA/transgelin (SM22) (A) and MYH11/calponin1 (CNN1) (B) were performed in 1 month AVFs. Scale bars = 50 μm. Fig. S4. VSMCs in common carotid artery were labeled with GFP in mTmG/SMMHC-ERCre+ mice after tamoxifen induction. Scale = 50 μm. [file 12967_2022_3727_MOESM1_ESM.pdf]

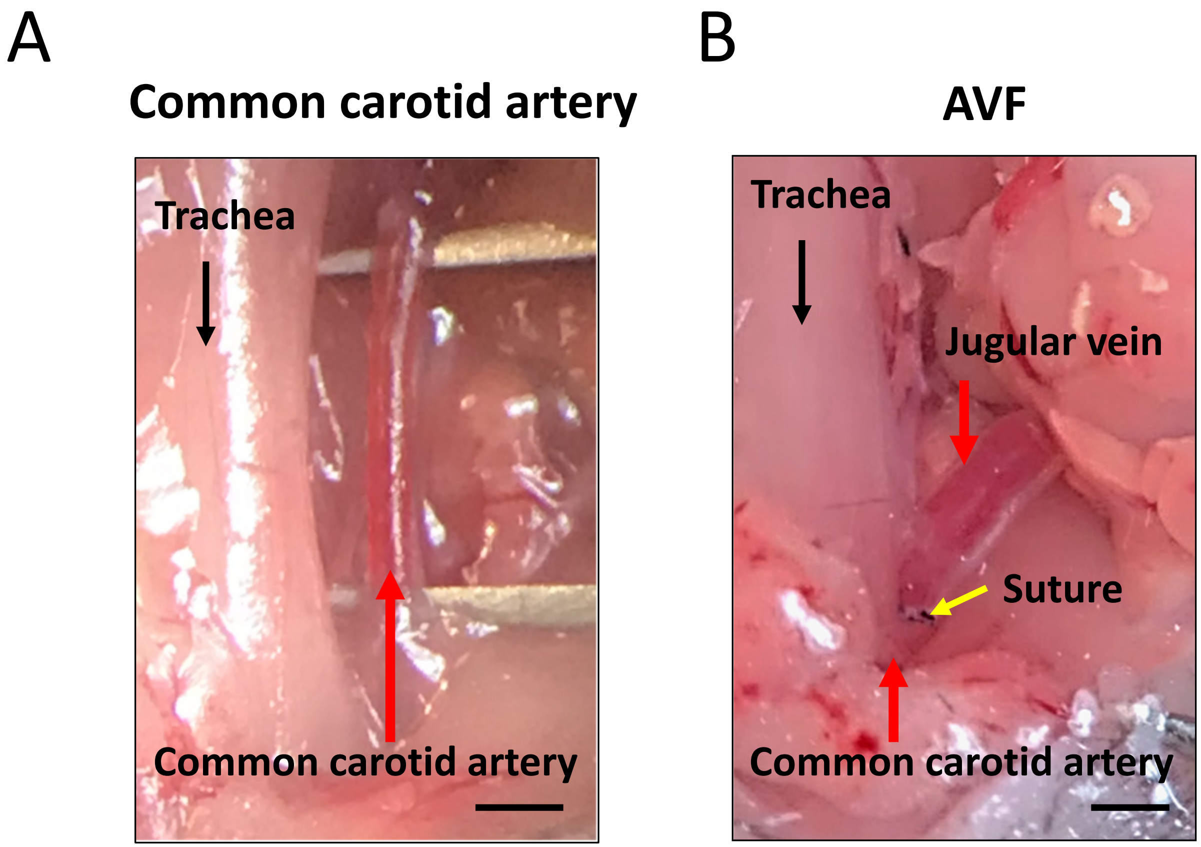

Supplemental figure 1

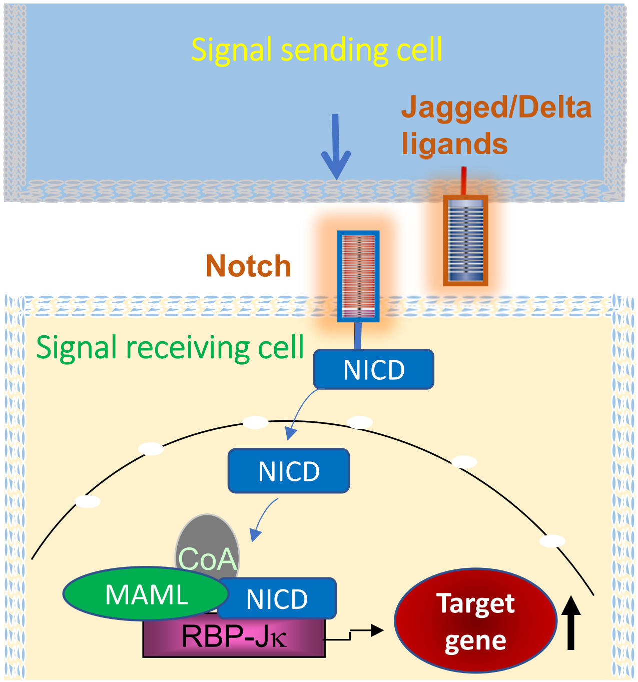

Supplemental figure 2

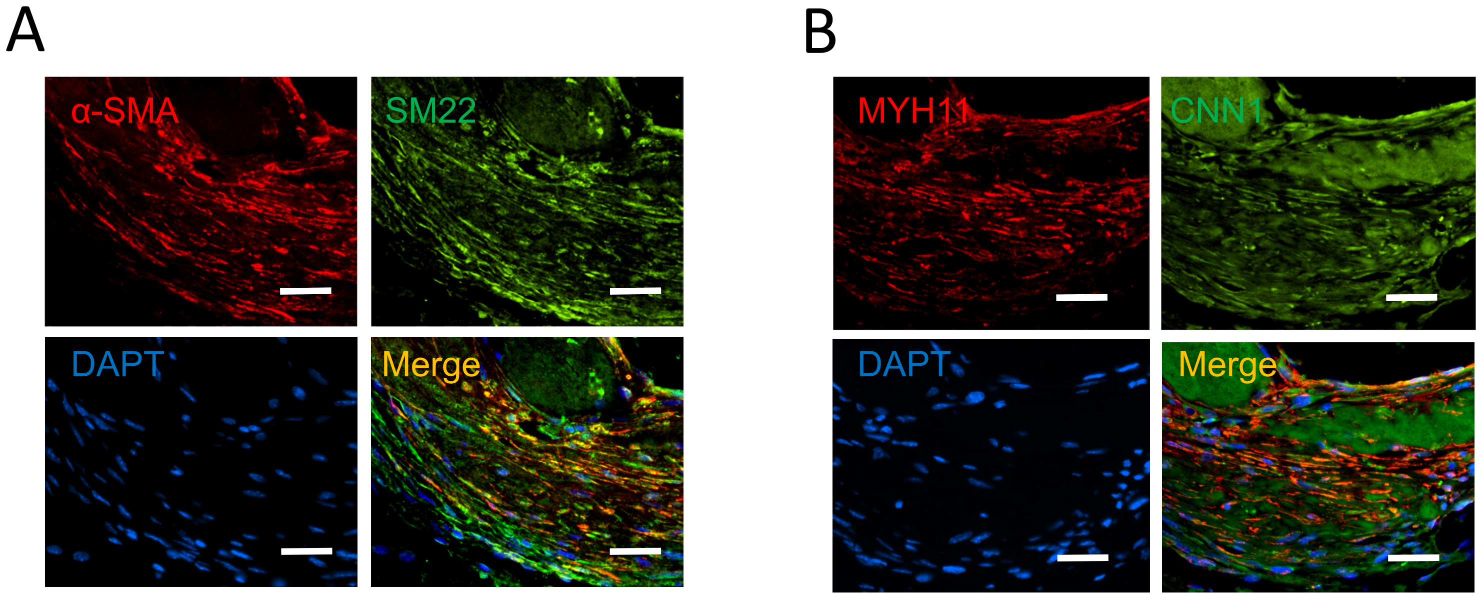

Supplemental figure 3

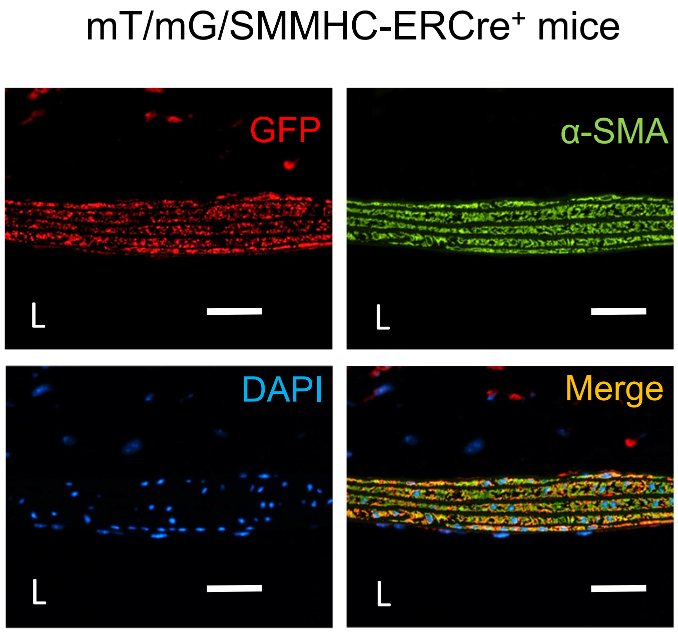

Supplemental figure 4
