# Supplementary material for: Temporal regulation of notch activation improves arteriovenous fistula maturation
Source: J Transl Med. 2022 Nov 23;20:543. doi: 10.1186/s12967-022-03727-7 (PMC9682688; doi:10.1186/s12967-022-03727-7)
Supplement: Supplementary file 2 — Additional file 2: Table S1. List of antibodies used in this study. [file 12967_2022_3727_MOESM2_ESM.pdf]

Supplemental Table

Table S1. List of antibodies used in this study

Antibody	company	Catalog number	Working dilution	Host
Calponin	Sigma	C2687	1 : 2000	Rabbit
α-SMA	Sigma	F4777	1 : 100	Mouse
α-SMA	Sigma	A5228	1 : 3200	Mouse
β-actin	Sigma	A5441	1 : 2000	Mouse
PCNA	Santa Cruz	sc-7907	1 : 500	Rabbit
ICAM	Santa Cruz	sc-71303	1 : 1000	Mouse
IL-1β	Santa Cruz	sc-32294	1 : 1000	mouse
Transgelin	Santa Cruz	sc-18513	1 : 200	Goat
GAPDH	Santra Cruz	sc-32233	1 : 1000	Mouse
PCNA	abcam	ab92552	1 : 500	Rabbit
α-SMA	abcam	ab5694	1 : 500	Rabbit
GFP	abcam	ab6556	1 : 2000	Rabbit
F4/80	abcam	ab6640	1 : 250	Rat
CD45	BD Parmingen	553076	1 : 400	Rat
VE-cadherin	BD Parmingen	550548	1 : 300	Rat
PECAM (CD31)	Cedarlane	DIA-310-M	1 : 50	Rat
Mac2	Cedarlane	CL8942AP	1 : 500	Rat
RBP-Jk	Cell Signaling	5313	1 : 500	Rabbit
Control IgG	Vector Labs	S-5000	1 : 200	Rabbit
